# Local recurrence of mammary Paget’s disease after nipple-sparing mastectomy and implant breast reconstruction: a case report and literature review

**DOI:** 10.1186/s12957-022-02746-4

**Published:** 2022-09-06

**Authors:** Qian Pu, Qianqian Zhao, Dezong Gao

**Affiliations:** 1 Department of General Surgery, Qilu Hospital (Qingdao), Cheeloo College of Medicine, Shandong University, 758 Hefei Road, Qingdao, Shandong China; 2 Department of Pathology, Qilu Hospital (Qingdao), Cheeloo College of Medicine, Shandong University, 758 Hefei Road, Qingdao, Shandong China

**Keywords:** Mammary Paget’s disease, Breast cancer, Nipple-sparing mastectomy, Local recurrence

## Abstract

**Objective:**

To provide a rare case of local recurrent Paget’s disease after nipple-sparing mastectomy (NSM) with immediate breast reconstruction with 10 years of disease-free survival and to analyze the clinical and pathological characteristics.

**Background:**

Mammary Paget’s disease can be considered a rare type of local recurrence after breast cancer treatment, both in cases of conservative surgery and NSM with immediate breast reconstruction (Lohsiriwat et al, Ann Surg Oncol 19:1850-1855, 2012). Recurrent patients who present with nipple-areolar Paget’s disease usually have unfavorable primary pathological characteristics and different latency periods. However, the recurrent status in patients with favorable primary pathological characteristics and the latency periods after NSM with immediate breast reconstruction are unclear.

**Methods:**

First, we present a case of local recurrent Paget’s disease in a young patient diagnosed with invasive breast carcinoma at age 30 who underwent NSM with primary silicone reconstruction. Then, the keywords “Paget’s disease” and “nipple-sparing mastectomy” were selected. Articles including the local recurrence of Paget’s disease after NSM were collected from the PubMed, Springer, and OVID databases, and the acquired relevant data were analyzed. We did not restrict our search by study design or publication date.

**Results:**

Five studies describing 31 cases of local recurrent Paget’s disease after NSM with implant breast reconstruction were included. The mean patient age reported was 45 years, and the average latency period from NSM to the local recurrence of Paget’s disease was 40.2 months. Recurrent tumor histological features were Paget’s disease with ductal carcinoma in situ (DCIS) in 16 patients (50%), Paget’s disease without DCIS in 13 patients (40.6%), and Paget’s disease with ductal intraepithelial neoplasia (DIN) in 3 patients (9.4%). The primary tumor histological feature was estrogen receptor (ER)(−)/progesterone receptor (PR)(−)/human epidermal growth factor receptor (HER-2)(+) in 21 patients (77.8%). Neither locoregional relapse nor metastatic events were found in these recurrent patients who accepted NAC excision after 4–5 years of follow-up. Our reported case showed that the patient experienced pregnancy and lactation after primary adjuvant chemotherapy and endocrine therapy. However, she developed an eczematoid lesion in the NAC 120 months after breast surgery. The histopathological examination was consistent with Paget’s disease of the breast. Complete NAC and breast silicone prosthesis removal were performed. The patient accepted no systematic or local therapy and is currently alive. It is noteworthy that the biological features of the primary tumor were ER(+), PR(+), and HER-2(−); however, the recurrent tumor changed to ER(−), PR(−), and HER-2(+).

**Conclusions:**

The local recurrence of Paget’s disease after NSM is uncommon; it may develop at a very early age and have a very long time to recurrence, as in our patient, who presented with recurrence 10 years after primary surgery. Surgeons should be wary of local recurrence of the nipple-areola complex after NSM in patients with ER-negative and HER-2-positive primary tumors. However, patients with ER/PR-positive and HER-2-negative tumors should not be neglected; we reported a case of an ER/PR-positive and HER-2-negative primary tumor, and ER-positive recurrent cases have the longest latency period. The local recurrence rate of Paget’s disease after NSM is low, and the prognosis is good in recurrent patients who accept further extensive NAC excision. Further systematic treatment was not considered for this patient.

## Backgrounds

Nipple-sparing mastectomy (NSM) is a surgical protocol designed to reduce the disabling psychological effects of radical mastectomy. The preservation of the nipple-areola complex (NAC) produces a more natural result of breast reconstruction, but it is suspected to increase local tumor recurrence consequent to occult nipple involvement [1]. Three years ago, Wu et al. reported that patients had a low incidence of cancer recurrence at the NAC after NSM with immediate breast reconstruction [2]. However, Han et al. indicated that the proper safety of NSM can be achieved only with a randomized clinical trial that compares NSM with non-NSM in patients where NSM is technically feasible [3]. The incidence rate of local tumor recurrence after NSM with preservation of the NAC is still unclear.

Mammary Paget’s disease (MPD) is a rare cancer of the nipple-areola complex. It is often associated with an underlying ductal in situ or invasive carcinoma [4]. After previous breast cancer treatment, MPD may be primary or present as a local recurrence [5]. Clinically, it manifests as a chronic pigmented, eczematous lesion with irregular borders in the nipple-areola complex. It is usually limited to the nipple or extended to the areola and may sometimes involve the surrounding skin [6, 7]. At present, there are few reports on the local recurrence of Paget’s disease after NSM.

Here, we report a patient with the local recurrence of mammary Paget’s disease who underwent NSM with primary silicone reconstruction 10 years ago. The age of onset was earlier than the average, and the latency time of recurrence was longer than the average. Local recurrence developed 10 years after the patient’s primary surgery.

## Materials and methods

### Nipple-sparing mastectomy operative technique

In our medical institution, Shandong University Qilu Hospital (Qingdao), the criteria for inclusion in the NSM study were as follows: patients with no clinical or radiological nipple involvement, patients with no inflammatory signs, and patients who had not undergone previous irradiation. Most importantly, a retro-areolar frozen section examination should be free of tumor cells. Patients with bilateral or locally advanced breast cancer, patients who had undergone prophylactic mastectomy, and patients who had undergone neoadjuvant radio- or chemotherapy were excluded. All patients provided informed consent before the operations.

The NSM skin incisions were designed mainly according to the tumor locations. An electric knife was used to perform glandular breast removal, and the optimum plane of dissection was the surgical plane just beneath the dermis. A retro-areolar frozen section was collected and examined intraoperatively in all cases. The NAC was preserved if the shape, color, and palpated features of the nipple were standard and when the NAC ducts were confirmed to be tumor-free in intraoperatively collected frozen biopsy samples. Sentinel lymph node biopsy and/or axillary lymph node dissection were performed. Prosthesis implantation is the most commonly used method for immediate breast reconstruction. Adjuvant systemic treatment was performed according to the current recommendations of the St. Gallen Consensus Conference and National Comprehensive Cancer Network guidelines.

### Literature search and analysis methods

This article was conducted with a systematic search strategy based on relevant literature available on PubMed, Springer, and OVID using the keywords “Paget’s disease” and “nipple-sparing mastectomy”, which were present in the abstracts and titles of the reviews. We did not restrict our search by study design or publication date. Five articles were screened [2, 8–11], and the literature search for the current study design included two sections: inclusion criteria and exclusion criteria. The inclusion criteria included English studies published in English, informative studies, and key searches, with no restriction for publication. The exclusion criteria included posters and editorials. The selected articles were reviewed carefully, and the data extracted were analyzed in terms of patient number and age at first breast cancer diagnosis, pathological features of the primary tumor, time to recurrence, conventional treatments, pathological features of recurrent tumors, and author name and publication year.

## Results

### Case report

A 30-year-old woman presented with a mass in the right upper quadrant of the breast in April 2010. The ultrasound image showed an irregular hypoechogenicity of 3.2 cm × 2.5 cm × 1.4 cm, with enlarged right axillary lymph nodes. The largest axillary node was 1.6 cm × 1.1 cm but was not evaluated with physical examination. Routine examination was performed, and no metastatic signs were found. The patient underwent core needle biopsy in the right breast mass, and pathological examination showed invasive ductal carcinoma. A large lymph node was biopsied with a fine needle, and no cellular components were found. The tumor had a histological grade of II, was strongly ER-positive (80%), strongly PR-positive (60%) and HER-2-negative, and had a Ki67 index of 30%. She refused a breast-conserving operation and underwent NSM with single-stage immediate reconstruction using a silicone prosthesis. The superficial and deep margins were free of carcinoma. Both frozen and serial sections revealed tumor-free retro-areolar resection margins. Unfortunately, sentinel lymph node biopsy failed, and axillary lymph node dissection revealed no metastases in 15 lymph nodes. Considering her tumor size and age, the patient accepted 4 cycles of concurrent TC chemotherapy (taxol/cyclophosphamide) and tamoxifen as adjuvant endocrine therapy because the invasive focus was strongly ER- and PR-positive and HER-2-negative.

**Fig. 1:**
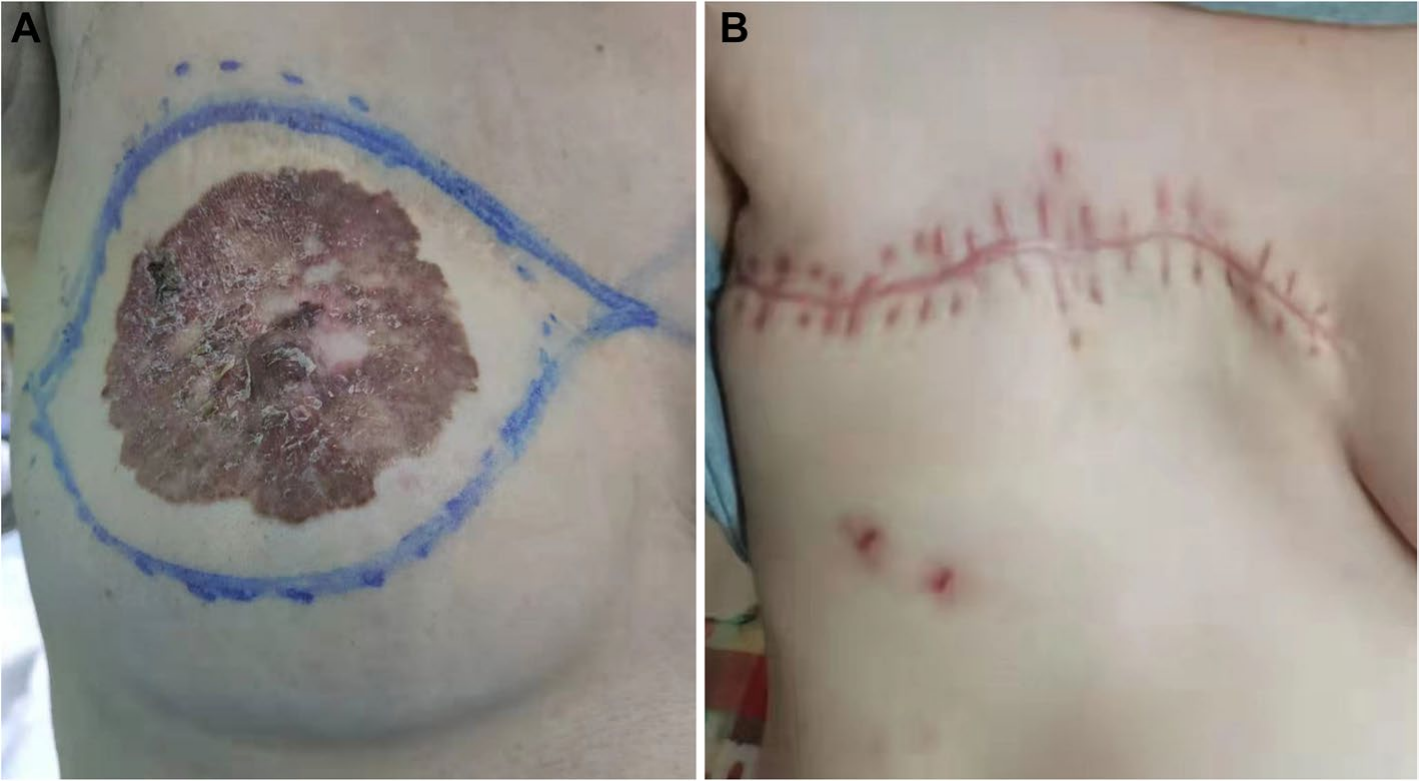
**A** Photograph taken by the breast surgeon when the patient visited the outpatient clinic 120 months after the NSM. **B** At the routine 3-month follow-up, physical examination revealed no abnormal findings

**Fig. 2:**
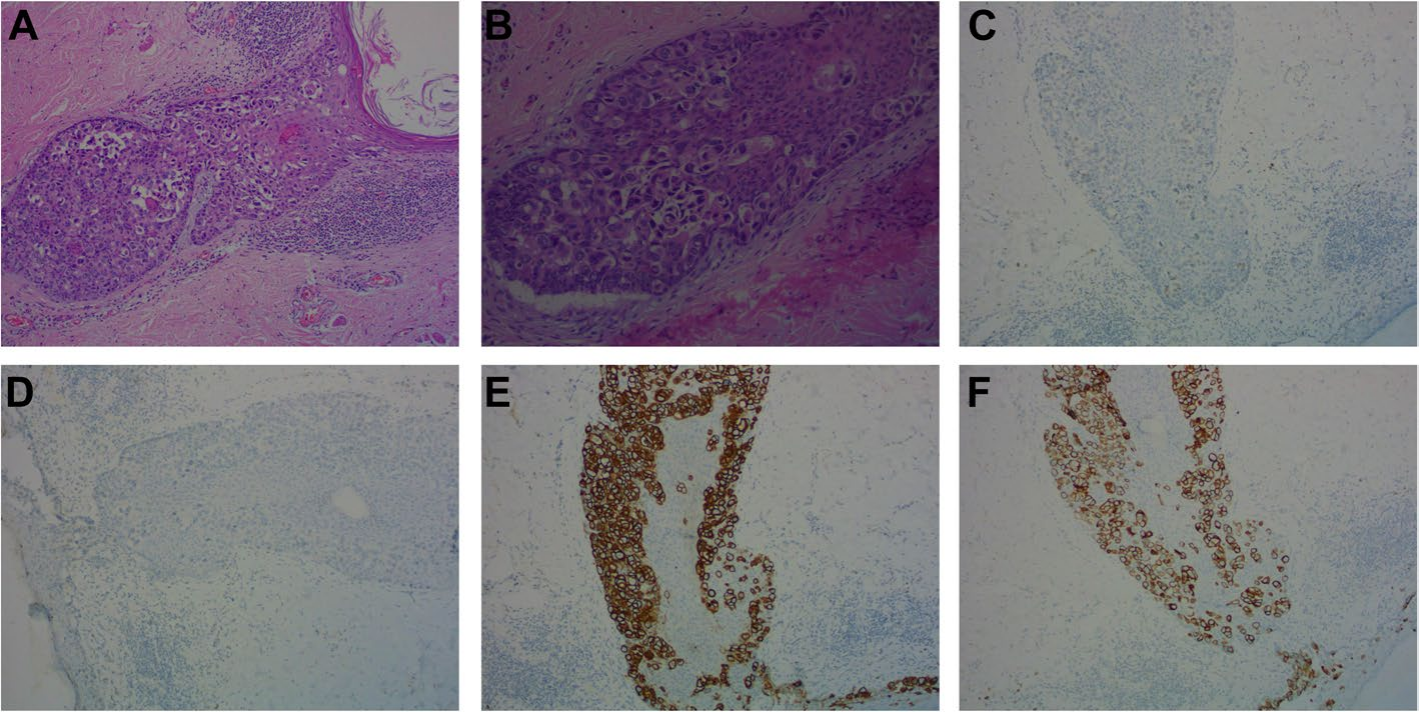
**A** Microscopic picture (H&E stain) revealed Paget’s disease with associated extensive DCIS (H&E, × 100 original magnification). **B** Paget’s disease characterized by infiltration of the epidermis by large tumor cells showing pale vacuolated cytoplasm and pleomorphic nuclei (H&E, × 100 original magnification). (CD) Negative estrogen receptor (ER) and progesterone receptor (PR) immunostaining in the Paget cells (× 100 original magnification). (EF) Her-2-positive cells and cytokeratin-7 (CK7)-positive cells constituting Paget’s disease (immunohistochemistry, × 100 original magnification)

At the routine 7-year follow-up, physical examination and breast ultrasound revealed no abnormal findings. The patient experienced pregnancy and had a history of breastfeeding during 2017–2019. However, approximately 120 months (2020) after NSM, the patient presented with an eczematoid lesion on the right nipple. There was a delay in treatment of nearly 1 year due to the COVID-19 pandemic. The lesion gradually increased in size and measured up to 8 cm × 7 cm at the outpatient clinic follow-up visit (Fig. 1A). Mammography and ultrasound were unremarkable. Staging scans showed no liver or lung metastasis, including a computed tomography scan of the thorax and abdomen. Bone scan showed no metastasis. Skin excision biopsy revealed Paget’s disease, and then a right NAC wide local excision and implant removal were performed (Fig. 1B). Pathological examination revealed Paget’s disease with extensive DCIS but no invasion (Fig. 2A, B). The entire area of the affected skin was excised, and the incision margins were free of tumor cells. What caught our attention was that the carcinoma had immunohistologically changed to ER- and PR-negative and HER-2- and CK-7-positive (Fig. 2C–F). Routine follow-up was carried out, and to date, the patient is alive, and neither locoregional relapse nor metastatic events have occurred (Fig. 1B).

#### Literature review

Here, we retrieved 31 cases of recurrent Paget’s disease after NSM published in the literature. Only two literature reports included many patients, and the others were case reports. In 2012, Visnu et al. performed an analysis of 861 patients who had undergone NSM and focused on the local recurrence of Paget’s disease. In the 861 patients, 36 had local recurrence (4.18%), and among these, 7 had local recurrent Paget’s disease (0.8%). The average latency period from NSM to the local recurrence of Paget’s disease was 32 months (range, 12–49 months) [8]. Wu et al. retrospectively analyzed 962 breasts of 944 invasive breast cancer patients who underwent NSM with immediate breast reconstruction. There were 39 cases (4.1%) of cancer recurrence at the NAC identified as the first event, and among these were 19 Paget’s disease cases with or without DCIS (1.98%) [2]. The mean patient age was 49 years, and the disease reoccurred after a mean period of 60.7 months.

The clinicopathological characteristics and outcomes of the patient in our case and published cases of local recurrent Paget’s disease after NSM with primary reconstruction are shown in Table 1. Including the case in our article, 32 cases have been reported from 1980 to the present. Recurrent tumor histological features were Paget’s disease with DCIS in 16 patients (50%), Paget’s disease without DCIS in 13 patients (40.6%), and Paget’s disease with DIN in 3 patients (9.4%). The median age of the patients at the time of diagnosis was 43.8 years (range, 20–53 years), and the median time from NSM surgery to recurrence of Paget’s disease in the reported cases was approximately 42.7 months (ranging from 12 months to 135 months). Five patients with unclear pathological features underwent removal of the primary tumor (NA), and a total of 27 patients had complete pathological results recorded. In these 27 patients, the primary tumor histological features were ER(−)/PR(−)/HER-2(+) in 21 patients (77.8%), ER(+)/PR(+)/HER-2(+) in 3 patients (11.1%), ER(−)/PR(−)/HER-2(−) in 1 patient (3.7%), ER(+)/PR(−)/HER-2(−) in 1 patient (3.7%), and ER(+)/PR(+)/HER-2(−) in 1 patient (3.7%). From the above data, we found that the HER-2(+) subtype was the most common pathological characteristic in primary tumors (88.9%). All 31 reported patients underwent NAC excision and were alive for 4–5 years of follow-up. Only in our case was the pathological character of the primary tumor changed from ER(+)/PR(+)/HER-2(−) to ER(−)PR(−)HER-2(+) after recurrence. The interval between NSM surgery and recurrence was 120 months, the longest latency period in ER-positive patients.

**Table 1:** Summary of reported cases of local recurrence as Paget’s disease after NSM with primary reconstruction [2, 8–11]

Authors	Number of patients reported	Patient		Primary tumor	TTR (months)	Recurrence tumor		Treatment
		No	Age (years)	ER/PR/Her-2		Histotype	ER/PR/Her-2	
Zhen-Yu Wu et al. [2]	19/944	1	40s	N/N/P	135	Paget	N/N/P	Excision
		2	30s	N/N/P	30	Paget	NA	Excision
		3	20s	N/N/P	35	Paget	N/N/P	Excision and RT
		4	40s	P/P/P	77	Paget	P/N/P	Excision
		5	30s	N/N/P	29	DCIS and Paget	N/N/P	Excision
		6	30s	N/N/P	43	DCIS and Paget	NA	Excision
		7	40s	N/N/P	61	DCIS and Paget	N/N/P	Excision
		8	40s	N/N/P	23	DCIS and Paget	N/N/P	Excision
		9	40s	P/N/N	25	DCIS and Paget	NA	Excision and HT
		10	30s	N/N/P	44	DCIS and Paget	N/N/P	Excision
		11	30s	N/N/P	24	DCIS and Paget	N/N/P	Excision
		12	30s	N/N/P	25	DCIS and Paget	N/N/P	Excision
		13	30s	N/N/P	23	DCIS and Paget	N/N/P	Excision
		14	40s	N/N/P	51	DCIS and Paget	N/N/P	Excision and RT
		15	30s	P/P/P	54	microDCIS + Paget	N/N/P	Excision and HT
		16	30s	N/N/P	36	DCIS and Paget	N/N/P	Excision, RT, CTx, Hercetin
		17	30s	N/N/P	18	DCIS and Paget	NA	Excision
		18	40s	P/P/P	35	DCIS and Paget	P/N/P	Excision
		19	30s	N/N/P	15	Paget	N/N/P	Excision
Shearman CP, et al. [9]	3/584	20	41	NA	18	Paget	NA	Excision
		21	51	NA	18	Paget	NA	Excision
		22	50	NA	48	Paget	NA	Excision
Mendez-Fernandez, et al. [10]		23	53	NA	96	Paget	NA	Excision
Harness JK, et al. [11]	1/43	24	NA	NA	34	Paget	NA	Excision
Lohsiriwat V et al. [8]	7/861	25	53	N/N/N	43	Paget and DIN	NA	Excision
		26	38	N/N/P	47	DCIS and Paget	NA	Excision and RT
		27	48	N/N/P	48	Paget	NA	Excision
		28	44	N/N/P	12	Paget and DIN	NA	Excision
		29	38	N/N/P	27	Paget	NA	Excision
		30	37	N/N/P	49	Paget and DIN	NA	Excision
		31	42	N/N/P	22	Paget	NA	Excision
Present study	1	32	30	P/P/N	120	DCIS and Paget	N/N/P	Excision and HT

*Abbreviations*: *DCIS* Ductal carcinoma in situ, *DIN* Ductal intraepithelial neoplasia, *ER* Estrogen receptor, *PR* Progesterone receptor, *N* Negative, *P* Positive, *NA* Not applicable, *TTR* Time to recurrence, *RT* Radiation therapy, *HT* Hormonal therapy, *CTx* Chemotherapy

## Discussion

Mammary Paget’s disease is characterized by neoplastic cells with epithelial features in the epidermal layer of the nipple-areolar complex. It is often associated with underlying breast cancer [12]. The pathological origin of Paget cells has been debated. The epidermotropic theory states that tumor cells migrate from an underlying breast carcinoma to the epidermis, but the opposing view advocates that Paget’s disease results from the malignant transformation of cells that are already present in the epidermis [13]. In our case, the clinical presentations of local recurrent Paget’s disease were not different from the primary Paget’s disease tumor reported in the literature. The most frequently reported symptoms are eczematous nipple discharge, crusting, scaling, bleeding, or ulceration. The eczema reaction starts at the nipple and then spreads to the areola and surrounding skin [14]. Mastectomy has been the standard treatment for mammary Paget’s disease.

NSM is characterized by preserving the entire nipple-areola complex and breast skin envelope despite removing mammary tissue [15]. Currently, NSM followed by immediate breast reconstruction has gained increased acceptance, with a growing emphasis on achieving excellent aesthetic results and improved quality of life without compromising oncological safety. Nevertheless, the application of NSM for breast cancer remains controversial because of the limited available data, including long-term follow-up data and accurate evaluations of modern therapeutic NSM. Currently, the main concern associated with NSM is the risk of local cancer recurrence at the retained NAC consequent to occult nipple involvement [1] Considering the fact that the breast size is relatively small in Chinese women, our indications for NSM with immediate breast reconstruction using silicone prostheses were as follows: a tumor size less than 5 cm in the largest diameter, earlier TNM stages, the absence of clinical swelling of the lymph nodes and a safe tumor-areola distance. The NAC was preserved if the palpation findings, shape, and color of the nipple were normal. Furthermore, we routinely performed intraoperative frozen biopsy examinations of the retro-areolar resection margins to determine nipple involvement. From the reported data, we found that the local NAC recurrence rate after NSM was approximately 4%, in which the local recurrence rate of Paget’s disease was 1–2%, and the prognosis was good in the recurrent patients who underwent extensive NAC excision.

Young age, ER negativity, and HER-2 positivity were significantly associated with a higher rate of cancer recurrence at the NAC in a univariate analysis [16]. Wu et al. believed that primary carcinomas with DIN or invasive ductal carcinomas with extensive in situ components, hormonal receptor negativity, a high pathological grade, and the HER-2-positive (non-luminal) subtype tend to be significantly associated with increased rates of local recurrent Paget’s disease [2]. Chen et al. also observed that all patients with local recurrent Paget’s disease had primary tumors that were ER/PR-negative and had a high pathological grade [5]. Caliskan et al. noticed that patients with Paget’s disease were more likely to have ER and PR negativity, high histological grades, and HER-2 positivity [17]. Considering the molecular profiles of these recurrent Paget’s disease patients, the HER-2-positive subtype (HER-2-positive, ER-negative, and PR-negative) was the most common type and expressed statistical significance compared with other types [18]. However, it is noteworthy that in our case, the patient had an ER-/PR-positive and HER-2-negative primary tumor and had the longest latency period in reported ER-positive recurrent cases. However, the immunochemical characteristics were changed to ER/PR negativity and HER-2 positivity. From the data reported by Wu et al. [2], the largest study of recurrent Paget’s disease, we found that the immunochemical characteristics did not change in recurrent tumors compared with primary tumors that expressed ER/PR-negativity and HER-2 positivity. Although there was one patient whose immunochemical characteristics changed from ER/PR-positive to ER/PR-negative, the HER-2-positive expression did not change [2]. These reported data suggest that unfavorable immunochemical characteristics may indicate Paget’s disease recurrence, but our case was exceptional.

Recurrence occurred 10 years after the first treatment and the tumor presented different pathological features; it looked more like a second primary cancer. Was this a local recurrence or a second primary cancer? The latest viewpoint is that the risk of breast cancer recurrence can last for 10 to 32 years [1]. Factors that are independently associated with a high risk of late recurrence include a diagnosis before the age of 40 years, ER-positive tumors, treatment with breast-conserving surgery, 4 or more positive lymph nodes, and a primary tumor diameter of 20 mm or greater. Among patients with ER-positive and HER-2-negative breast cancer, whose prognoses were good, at least half of them experienced recurrence 5 years after the initial diagnosis [19]. Five to 20 years after the initial diagnosis, the risk of distant recurrence ranged from 13 to 41%, depending on the characteristics and lymph node status of the primary tumor, and a larger tumor size and higher histological grade were associated with an increased risk of recurrence [20]. Recurrences occurred even up to 32 years after the initial diagnosis [21]. The patient we reported was only 30 years old at the time of initial diagnosis, with a tumor diameter greater than 3 cm. These factors were independently related to a high risk of recurrence. The good immunochemical characteristics in our patient may be the reason for her long recurrent latency period, so we consider local recurrence to have occurred. In addition, adjuvant endocrine therapy for breast cancer is currently recommended to extend to 10 years [22]. Our patient had a history of pregnancy and lactation during treatment, and we think that changes in hormone levels and the interruption of endocrine therapy might have contributed to the local recurrence of Paget disease. Although a second attempt with an implant has a high success rate after failed prosthetic breast reconstruction due to infection or exposure [23], our reported patient was not an appropriate candidate because she did not have enough of a skin envelope after extensive NAC excision. The desire for secondary autologous reconstruction was strong in patients with failed implants caused by infection or exposure [23]. Our patient refused secondary autologous reconstruction because of tumor recurrence and worried about recurrence again.

## Conclusions

Currently, more young breast cancer patients undergo NSM, but the local recurrence of Paget’s disease can be seen after subcutaneous mastectomy and reconstruction. To our knowledge, only 32 cases of recurrent nipple areolar Paget disease have been reported in the literature, including our reported patient with NSM reconstruction, since 1980. The local NAC recurrence rate after NSM is low, and the local recurrence rate of Paget’s disease is only 1–2%. Furthermore, the prognosis is good in recurrent patients who undergo extensive NAC excision. Therefore, NSM with prosthetic breast reconstruction is a safe surgical method for young patients with early breast cancer, but long-term postoperative monitoring is required to identify local recurrence, especially in patients with ER- and PR-negative and HER-2-positive tumors.

## Data Availability

The data sets used and/or analyzed during the current study are available from the corresponding author on reasonable request.

## Abbreviations

NSM: Nipple-sparing mastectomy
DCIS: Ductal carcinoma in situ
NAC: Nipple-areola complex
MPD: Mammary Paget’s disease
ER: Estrogen receptor
PR: Progesterone receptor
DIN: Ductal intraepithelial neoplasia
